# Multimodality Imaging in Aldosterone-Induced Cardiomyopathy: Early Detection and Prognostic Implications

**DOI:** 10.3390/diagnostics15151896

**Published:** 2025-07-29

**Authors:** Francesca Zoccatelli, Gabriele Costa, Matteo Merlo, Francesca Pizzolo, Simonetta Friso, Luigi Marzano

**Affiliations:** Unit of Internal Medicine B, Department of Medicine, University of Verona School of Medicine, Azienda Ospedaliera Universitaria Integrata Verona, Policlinico “G.B. Rossi”, 37134 Verona, Italy

**Keywords:** primary aldosteronism, echocardiography, left ventricular hypertrophy, cardiac remodeling, adrenalectomy, mineralocorticoid receptor antagonists, cardiac magnetic resonance

## Abstract

Primary aldosteronism (PA), the most common cause of secondary hypertension, is increasingly recognized as an independent driver of adverse cardiac remodeling, mediated through mechanisms beyond elevated blood pressure alone. Chronic aldosterone excess leads to myocardial fibrosis, left ventricular hypertrophy, and diastolic dysfunction via mineralocorticoid receptor activation, oxidative stress, inflammation, and extracellular matrix dysregulation. These changes culminate in a distinct cardiomyopathy phenotype, often underrecognized in early stages. Multimodality cardiac imaging, led primarily by conventional and speckle-tracking echocardiography, and complemented by exploratory cardiac magnetic resonance (CMR) techniques such as T1 mapping and late gadolinium enhancement, enables non-invasive assessment of structural, functional, and tissue-level changes in aldosterone-mediated myocardial damage. While numerous studies have established the diagnostic and prognostic relevance of imaging in PA, several gaps remain. Specifically, the relative sensitivity of different modalities in detecting subclinical myocardial changes, the long-term prognostic significance of imaging biomarkers, and the differential impact of adrenalectomy versus medical therapy on cardiac reverse remodeling require further clarification. Moreover, the lack of standardized imaging-based criteria for defining and monitoring PA-related cardiomyopathy hinders widespread clinical implementation. This narrative review aims to synthesize current knowledge on the pathophysiological mechanisms of aldosterone-induced cardiac remodeling, delineate the strengths and limitations of existing imaging modalities, and critically evaluate the comparative effects of surgical and pharmacologic interventions. Emphasis is placed on early detection strategies, identification of imaging biomarkers with prognostic utility, and integration of multimodal imaging into clinical decision-making pathways. By outlining current evidence and highlighting key unmet needs, this review provides a framework for future research aimed at advancing personalized care and improving cardiovascular outcomes in patients with PA.

## 1. Introduction

Primary aldosteronism (PA) is recognized as the most common cause of secondary hypertension, with a prevalence reaching up to 10% in hypertensive populations and over 20% in those with resistant hypertension [1,2,3,4,5,6]. Beyond its hypertensinogenic effects, PA exerts profound cardiovascular toxicity, contributing to left ventricular hypertrophy (LVH), myocardial fibrosis, atrial remodeling, and an increased risk of heart failure and arrhythmias [5,7,8,9]. These adverse outcomes occur not merely due to elevated blood pressure, but also through direct, non-hemodynamic effects of aldosterone on cardiac tissue via mineralocorticoid receptor (MR) activation, oxidative stress, inflammatory pathways, and extracellular matrix remodeling [10,11,12].

The cardiovascular burden of PA is increasingly attributed to its distinct cardiac phenotype, which we might define as ‘aldosterone-induced cardiomyopathy’. This condition is characterized by disproportionate LV mass increase relative to blood pressure levels, frequent diastolic dysfunction, and subclinical systolic impairment detectable by advanced imaging modalities such as speckle-tracking echocardiography and cardiac magnetic resonance imaging (CMR). Importantly, these myocardial changes are at least partially reversible with specific treatment [13].

Current therapeutic strategies for PA include unilateral adrenalectomy, typically indicated in cases of unilateral PA, and medical therapy with MR antagonists (MRAs), such as spironolactone and eplerenone, used primarily for bilateral adrenal hyperplasia. Both modalities aim to normalize aldosterone levels and mitigate its downstream effects [14,15,16]. However, accumulating evidence suggests that adrenalectomy results in more pronounced reductions in left ventricular mass and greater improvements in cardiac geometry and function than MRA therapy, even when biochemical control is achieved [17,18,19]. Despite the growing recognition of this phenomenon, current meta-analyses and systematic reviews of the literature continue to consider surgical and medical treatments comparable in reversing cardiac mass and left ventricular hypertrophy [15,16,20,21,22], largely due to limited randomized evidence directly comparing their cardiovascular outcomes, and the insufficient number and statistical power of existing investigations [23,24,25,26,27,28,29].

This disconnect underscores the urgent need for a mechanistic and imaging-based reappraisal of the divergent effects of adrenalectomy and medical therapy on cardiac remodeling in PA. In this narrative review, we aim to synthesize the evolving evidence on PA-induced cardiac remodeling and its differential reversibility following surgical versus pharmacological treatment. By integrating pathophysiological insights, advanced echocardiographic and CMR imaging data, and clinical outcome studies, we explore the growing premise that adrenalectomy, particularly in patients with unilateral PA, may offer superior regression of left ventricular hypertrophy and restoration of cardiac architecture. In this way, we invite a rethinking of current paradigms and a closer examination of why adrenalectomy may represent a more compelling therapeutic strategy for patients with unilateral PA, potentially offering a path toward more effective cardiovascular protection.

## 2. Aldosterone Effects on the Heart

**Figure 1:**
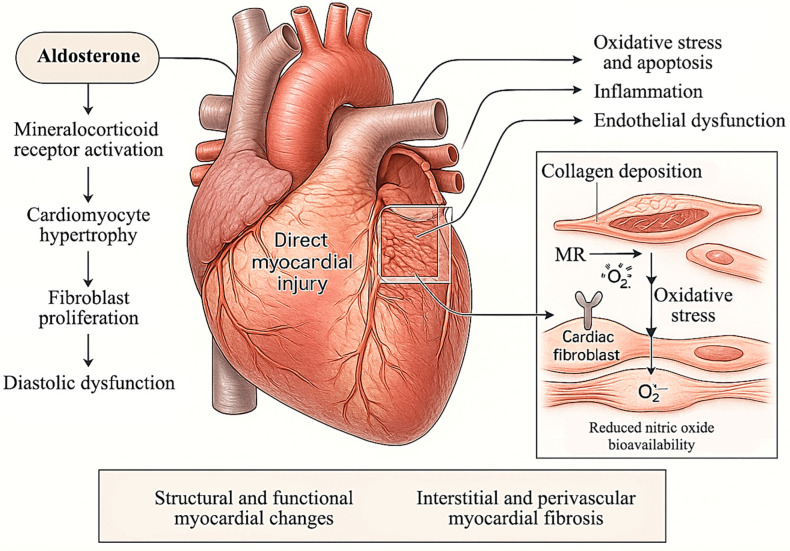
**Structural and Cellular Mechanisms of Aldosterone-Induced Cardiac Remodeling.** This illustration demonstrates the complex pathological mechanisms by which aldosterone excess drives cardiac remodeling in primary aldosteronism. Key features include cardiomyocyte hypertrophy, apoptosis, interstitial and perivascular fibrosis, and microvascular remodeling. These changes are mediated through mineralocorticoid receptor activation and amplified by oxidative stress, inflammation, and dysregulated extracellular matrix turnover. Collectively, these alterations contribute to impaired myocardial relaxation, diastolic dysfunction, and adverse ventricular remodeling. **Abbreviations:** MR, mineralocorticoid receptor.

Aldosterone exerts potent deleterious effects on cardiovascular structure and function, extending well beyond its canonical role in sodium retention and blood pressure regulation [5,30]. In the setting of primary aldosteronism (PA), chronic exposure to inappropriately elevated aldosterone levels particularly in the context of suppressed renin, drives direct and indirect myocardial injury through mechanisms that are both hemodynamic and non-hemodynamic in nature [9,10]. At the cellular level, aldosterone promotes cardiomyocyte hypertrophy and apoptosis, stimulates fibroblast proliferation, and enhances collagen deposition, culminating in interstitial and perivascular myocardial fibrosis (Figure 1) [31,32,33]. These effects are predominantly mediated via mineralocorticoid receptor (MR) activation in cardiomyocytes and cardiac fibroblasts, and are further amplified by mechanisms driven by oxidative stress, inflammation, and endothelial dysfunction. Notably, aldosterone-induced oxidative stress contributes to increased expression of profibrotic genes and reduced nitric oxide bioavailability, thereby impairing myocardial relaxation and promoting diastolic dysfunction [9,34].

Experimental models and clinical studies have consistently demonstrated that the myocardial damage associated with aldosterone excess occurs independently of blood pressure, underscoring a primary toxic effect on the myocardium. This is supported by histopathological analyses showing diffuse fibrosis and microvascular remodeling in aldosterone-treated animals and in patients with PA, even when matched for blood pressure values with essential hypertensive controls [9,34]. In addition, aldosterone disrupts extracellular matrix turnover by inhibiting matrix metalloproteinases and stimulating their tissue inhibitors, thereby promoting fibrotic remodeling. These structural changes translate clinically into altered ventricular geometry, reduced compliance, and impaired filling pressures, hallmarks of the cardiomyopathy associated with PA [29,35,36].

Importantly, the myocardial consequences of aldosterone are not uniform across all individuals. The extent and pattern of cardiac remodeling likely depend on the intensity and duration of aldosterone excess, genetic predispositions (such as KCNJ5 mutations), dietary sodium intake, and the presence of coexisting conditions such as diabetes mellitus or cortisol co-secretion [28,37]. Therefore, in PA, aldosterone functions as a central pathophysiological driver of cardiac remodeling, not merely as a surrogate of elevated blood pressure. The early identification and targeted treatment of aldosterone excess, especially through adrenalectomy in unilateral forms, are thus crucial for mitigating myocardial injury and preventing irreversible structural damage.

## 3. Evolution of Echocardiography in Primary Aldosteronism

**Figure 2:**
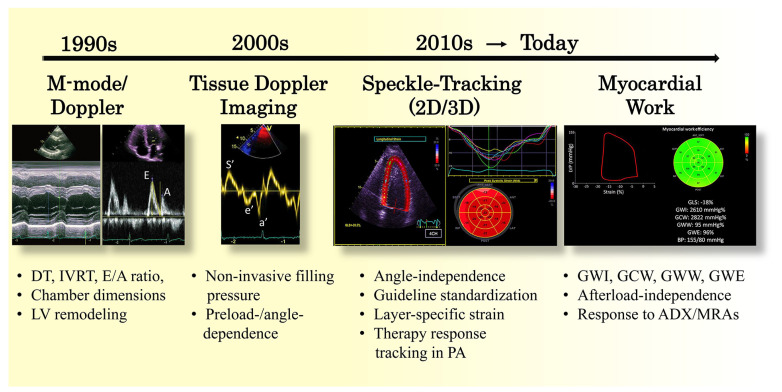
**Timeline of Echocardiography Progress in Primary Aldosteronism. 1990s**—M-mode and Doppler: Detection of LV hypertrophy, altered diastolic filling (E/A, DT, IVRT). **2000s**—Tissue Doppler Imaging (TDI): Early diastolic mitral annular velocity (e′), filling pressure (E/e′). **2010s**—Speckle-Tracking Echocardiography: Global longitudinal strain (GLS) for early systolic dysfunction. Myocardial Work: Afterload-independent assessment of myocardial performance. **Today**—Integrated Imaging: Comprehensive assessment of LV structure, systolic and diastolic function in PA. **Abbreviations:** DT, deceleration time; IVRT, isovolumic relaxation time; E/A ratio, early (E) to late (A) transmitral flow velocities; LV, left ventricle; S′, systolic annular velocity; e′, early diastolic mitral annular velocity; a′, late diastolic mitral annular velocity; GLS, global longitudinal strain; GWI, global work index; GCW, global constructive work; GWW, global wasted work; GWE, global work efficiency; ADX, adrenalectomy; MRAs, mineralocorticoid receptor antagonists.

Echocardiography in primary aldosteronism (PA) has evolved considerably, advancing from rudimentary M-mode and Doppler assessments in the 1990s to today’s sophisticated tissue and strain imaging techniques (Figure 2). Early human studies by Rossi et al. (1996–1997) demonstrated that PA patients, particularly those with Conn’s adenoma, exhibit pronounced left ventricular (LV) hypertrophy and impaired diastolic filling, even when compared to blood pressure-matched essential hypertensives [24,38]. These observations were consistent with earlier findings by Suzuki et al., who highlighted distinct LV structural abnormalities in PA compared to unilateral renovascular hypertension, underscoring the unique cardiac remodeling associated with aldosterone excess [39]. Simultaneously, animal studies confirmed that chronic aldosterone exposure promotes diffuse myocardial fibrosis via mineralocorticoid receptor-mediated collagen deposition [40,41,42]. These data framed the concept of an ‘aldosterone cardiomyopathy’ and motivated echo-based screening for subclinical dysfunction. Initially, echo parameters focused on transmitral Doppler (E/A ratio, deceleration time [DT], isovolumic relaxation time [IVRT]), and chamber dimensions. By the early 2000s, Doppler tissue imaging (TDI) enabled quantification of mitral annular velocities (e′) and estimation of filling pressures through the E/e′ ratio [43,44]. In the 2010s, two-dimensional speckle-tracking echocardiography revolutionized myocardial mechanics assessment, with global longitudinal strain (GLS) rapidly adopted as a sensitive marker of early systolic dysfunction, now endorsed by international guidelines [45,46,47,48]. More recently, the development of myocardial work analysis, based on non-invasive LV pressure–strain loops derived from echocardiography, has provided novel, load-adjusted insights into LV performance [49,50]. In PA cardiomyopathy, this technique has revealed increased global wasted work and reduced myocardial work efficiency, offering an integrated, quantitative assessment of both mechanical and energetic abnormalities that precede overt systolic dysfunction [51,52]. These findings have strengthened the role of myocardial work indices as emerging tools for characterizing PA cardiomyopathy.

## 4. Diastolic Function Parameters

Classic diastolic Doppler indices were first applied in primary aldosteronism (PA): an early study found the transmitral E/A ratio (and E wave integral) to be significantly lower in PA than in essential hypertension (EH), with a compensatory rise in atrial filling [38,53]. These findings implied higher LV stiffness in PA. However, Doppler E/A and deceleration time (DT) are age- and load-dependent and often normalize in comparison studies [54]. For example, in a recent propensity score-matched cohort study, no significant differences were observed in mean E/A ratio or deceleration time (DT) between patients with aldosterone-producing adenoma (APA) and those with essential hypertension (EH) [55]. By contrast, Doppler tissue imaging (TDI)-derived measures prove more sensitive: PA patients consistently show reduced e′ velocity (slower relaxation) and elevated E/e′ ratios (higher filling pressures) compared to EH [55,56,57]. Importantly, these TDI abnormalities improve after surgical cure, supporting their aldosterone link. Left atrial (LA) volume, a chronic diastolic load marker, is also often enlarged in PA [56]. In one series, APA patients had markedly higher indexed LA volume than those with idiopathic hyperplasia (≈40 vs. 30 mL/m^2^) [58]. Pulmonary artery pressure estimated from the tricuspid-regurgitation (TR) jet remains under-investigated in primary aldosteronism, likely because frank pulmonary hypertension only emerges in advanced diastolic dysfunction. Nonetheless, Chen et al. have shown that PA patients exhibit significantly higher TR-derived systolic pulmonary artery pressures than essential hypertensives, even though both groups’ values fall within conventionally accepted normal ranges [59].

## 5. Systolic Function Parameters (Speckle Tracking)

In primary aldosteronism (PA), conventional measures of systolic function, such as left ventricular ejection fraction (LVEF) or fractional shortening, are typically preserved; therefore, early systolic impairment remains subclinical [55]. Speckle-tracking echocardiography (STE) has consequently emerged as a sensitive tool for detecting subtle, subclinical myocardial dysfunction. Multiple studies have consistently reported significantly impaired left ventricular global longitudinal strain (GLS) in patients with PA compared to those with essential hypertension (EH) or normotensive controls. For example, Chen et al. demonstrated a mean GLS of approximately −17.8% in PA patients versus −20.3% in EH subjects (*p* < 0.001), despite similar LVEF values across groups [47]. Furthermore, layer-specific STE has revealed a characteristic transmural strain gradient in PA, with the most pronounced reduction observed in endocardial longitudinal and circumferential strain. Specifically, patients with aldosterone-producing adenomas (APAs) exhibited endocardial GLS values of approximately −20%, compared to −24% in EH subjects [48]. This progressive decline in strain from the endocardium to the epicardium reflects the layered vulnerability of myocardial fibers to aldosterone-induced injury.

Importantly, STE-derived strain parameters provide a quantitative assessment of systolic dysfunction that correlates with biochemical severity, particularly circulating aldosterone levels, even when conventional LVEF and regional wall motion appear normal.

## 6. Comparative Echocardiographic Findings (PA vs. EH vs. Controls)

High-quality comparative studies consistently demonstrate that primary aldosteronism (PA) is associated with more pronounced cardiac remodeling than either essential hypertension (EH) or normotension. In propensity score-matched cohorts, patients with aldosterone-producing adenoma (APA) exhibit significantly higher left ventricular (LV) mass index, lower diastolic mitral annular velocity (e′), and elevated E/e′ ratios compared to EH subjects [55]. Similarly, Cesari et al. reported that both PA and secondary hyperaldosteronism are associated with substantially greater LV hypertrophy and diastolic dysfunction than those observed in healthy controls [57]. This is further supported by tissue Doppler studies, which revealed a diastolic dysfunction prevalence of approximately 35% in PA, compared to near 0% in normotensive individuals [57]. Concordant findings have been reported with speckle-tracking echocardiography, where PA patients consistently demonstrate reduced global longitudinal strain (GLS) relative to those with EH [47]. Notably, the degree of GLS impairment correlates strongly with the biochemical severity of disease, particularly the aldosterone-to-renin ratio. Collectively, decades of research confirm that advanced echocardiographic modalities, including tissue Doppler imaging and speckle-tracking echocardiography, reliably detect more severe diastolic dysfunction and subtle systolic impairment in PA compared to EH and normotensive controls.

## 7. Echocardiographic and Imaging Markers of PA−Cardiomyopathy

Primary aldosteronism (PA) induces a distinct form of cardiomyopathy, characterized by concentric left ventricular remodeling and a specific pattern of both diastolic and systolic dysfunction, markedly divergent from the classical cardiac damage observed in essential hypertension (Table 1) [60,61]. This PA−cardiomyopathy phenotype arises largely from aldosterone-induced myocardial fibrosis and hypertrophy, disproportionately exceeding what would be expected from blood pressure alone [10]. These alterations can be accurately captured using multimodal imaging, beginning with echocardiography as the first-line tool, and complemented by cardiac magnetic resonance (CMR) for tissue characterization [8,62].

Diastolic dysfunction in patients with PA arises primarily from aldosterone-induced myocardial fibrosis rather than from pressure-overload hypertrophy. This is supported by the observation of biventricular diastolic abnormalities in PA patients compared to sex-matched individuals with primary hypertension, even after adjustment for confounding variables [29]. Key echocardiographic findings include inversion of the early (E) to late (A) transmitral Doppler velocity ratio (E/A ratio < 1) and an elevated tissue Doppler E/e′ ratio, indicating increased reliance on atrial contraction for ventricular filling [38,55,57]. These functional changes are often accompanied by atrial enlargement and PQ interval prolongation, highlighting atrial electromechanical alterations and the development of atrial fibrillation risk [38,63,64].

A hallmark of PA is the disproportionate increase in left ventricular mass relative to afterload, suggesting a hemodynamically independent myocardial response. This concept is reinforced by differentiating between predicted left ventricular mass index (pLVMI) and inappropriate excess left ventricular mass index (ieLVMI), with the latter serving as a surrogate marker for aldosterone-induced non-hemodynamic remodeling [10,34].

While diastolic impairment is more prominent, early systolic dysfunction in PA is clinically relevant and often underrecognized. Ejection fraction (EF) may remain normal or even supra-normal due to hypervolemia, thereby masking contractile abnormalities. However, speckle-tracking echocardiography (STE) allows for early detection of subclinical systolic dysfunction by quantifying reductions in global longitudinal strain (GLS), which correlates with histological fibrosis and predicts adverse cardiac remodeling [65]. GLS abnormalities in PA can precede overt EF decline and serve as an early dynamic biomarker for treatment response, especially in patients with prolonged hypertension exposure, where reversibility is time-dependent. This reinforces the critical role of early diagnosis and timely intervention to prevent irreversible cardiac injury [7,10,21,66].

In this context, myocardial work analysis derived from pressure–strain loop modeling has emerged as a valuable echocardiographic tool, offering load-independent metrics of myocardial performance. A pivotal 2024 study by Chen et al. confirmed that patients with PA exhibit significantly elevated global wasted myocardial work and reduced myocardial work efficiency, which were independently associated with myocardial fibrosis as validated by CMR extracellular volume fraction and native T1 mapping. This highlights the potential of non-invasive myocardial work indices not only for detecting early subclinical systolic impairment but also for inferring myocardial fibrosis burden in PA, with comparable diagnostic accuracy to CMR-based assessments [51].

Transthoracic echocardiography (TTE) remains a widely accessible and cost-effective modality for serial cardiovascular assessment. It enables real-time quantification of cardiac geometry (e.g., LV mass, left atrial volume) and function, without exposure to contrast agents or radiation. Advanced techniques such as tissue Doppler imaging (TDI) and speckle-tracking echocardiography (STE) have been rigorously validated against invasive reference standards and are supported by established normative values across diverse populations [67,68,69,70].

Nevertheless, inherent limitations of TTE persist. Doppler and TDI parameters are angle- and preload-dependent, requiring stringent acquisition standards for consistent interpretation. For example, mitral inflow and annular velocities (e.g., E, e′, e′/a′) are significantly influenced by preload status, such as during ultrafiltration in dialysis, highlighting the load-sensitivity of these measures [71]. For instance, the E/e′ ratio, commonly used to estimate left ventricular (LV) filling pressures, can be elevated abnormally in obesity, where altered myocardial mechanics skew tissue Doppler-derived velocities [72,73], or in the presence of mitral annular calcification, which limits annular excursion and leads to falsely increased E/e′ values [74]. Similarly, the accuracy of speckle-tracking-derived strain analysis is contingent upon image quality and software-specific algorithms, which impairs inter-vendor comparability [75]. Furthermore, echocardiography cannot directly quantify myocardial fibrosis and relies on surrogate indicators such as wall thickness or strain abnormalities. Conventional Doppler indices (e.g., E/A ratio, IVRT, DT) lack sensitivity in early disease and can be confounded by age or rhythm disturbances.

Despite these caveats, expert-performed TTE remains the cornerstone for early detection of aldosterone-induced cardiac remodeling, offering integrative insights into LV geometry, function, and hemodynamics before the onset of symptomatic heart failure.

Cardiac magnetic resonance (CMR) imaging offers unmatched precision in the structural and tissue-level assessment of PA-cardiomyopathy (Table 1). Late gadolinium enhancement (LGE) reveals a characteristic mid-wall, patchy non-ischemic fibrosis pattern, typically in the interventricular septum or basal inferolateral segments [8,76]. Quantitative T1 mapping and extracellular volume (ECV) quantification allow for the detection of diffuse interstitial fibrosis, which correlates with aldosterone exposure and may guide therapy optimization. Beyond its diagnostic power, CMR provides high reproducibility and low operator dependence, and serves as an excellent tool for longitudinal monitoring in patients undergoing adrenalectomy or mineralocorticoid receptor antagonist therapy [8]. Importantly, comparative studies, including those utilizing semiautomated volumetric techniques, have consistently confirmed a systematic underestimation of left ventricular (LV) volumes by transthoracic echocardiography compared to cardiac magnetic resonance (CMR), thereby reinforcing their complementary roles in clinical practice [77]. Beyond methodological discrepancies, CMR-based investigations have provided novel insights into the cardiac remodeling phenotype associated with PA. In a prospective study, Wu et al. demonstrated that patients with PA exhibit significantly higher indexed LV end-diastolic and end-systolic volumes, as well as increased LV mass and native T1 values, compared to both essential hypertension patients and healthy controls, suggesting a distinct pattern of eccentric hypertrophy and diffuse myocardial fibrosis detectable only through advanced CMR techniques [78]. Consistent with these findings, Higuchi et al. reported that patients with aldosterone-producing adenoma (APA), display significantly elevated LV volumetric indices and native T1 values relative to those with bilateral hyperaldosteronism, even after adjustment for confounding variables [79]. These findings underscore the role of aldosterone excess not only in hypertrophic remodeling but also in promoting volume overload and myocardial tissue injury, particularly in APA, where the hormonal burden is often more pronounced. Furthermore, high-resolution speckle-tracking echocardiography and feature-tracking CMR offer synergistic insights into LV mechanics and myocardial deformation, reinforcing the added value of integrating both modalities for comprehensive myocardial phenotyping in PA-cardiomyopathy [80,81]. The incorporation of multimodality imaging into clinical workflows facilitates a more refined and complete definition of PA-induced cardiac remodeling and strengthens the rationale for individualized, mechanism-targeted therapeutic strategies.

## 8. Discussion

Primary aldosteronism (PA) represents a paradigm of potentially curable secondary hypertension with disproportionately high cardiovascular morbidity and mortality, largely driven by aldosterone-mediated structural and functional cardiac remodeling [38,82,83,84]. This review underscores the critical concept that PA-induced cardiomyopathy is not merely a consequence of blood pressure elevation but is characterized by specific myocardial alterations, including left ventricular hypertrophy (LVH), diastolic dysfunction, and diffuse myocardial fibrosis, all of which may occur independently of systemic hemodynamic load [10,38]. These pathophysiological derangements are largely attributable to the profibrotic, proinflammatory, and oxidative effects of excess aldosterone on myocardial tissue, particularly in the absence of adequate mineralocorticoid receptor (MR) blockade or adrenalectomy [9,11].

Cumulative data strongly support the superior efficacy of unilateral adrenalectomy in patients with lateralized disease, not only in achieving biochemical remission but also in driving more profound and durable regression of LVH compared to MR antagonist therapy [19,85,86]. Although various prognostic scores have been developed to predict complete clinical success following adrenalectomy [87,88,89,90], and despite the fact that only a subset of patients achieve full resolution of hypertension, surgical intervention remains uniquely effective [86]. By directly removing the source of aldosterone excess, adrenalectomy induces a swift and sustained decline in circulating hormone levels, promotes early hemodynamic unloading of the heart, and more potently disrupts profibrotic molecular pathways compared to medical therapy [13,21]. By contrast, although MR antagonists such as spironolactone and eplerenone have demonstrated beneficial effects on cardiac structure and function, their impact is modulated by dosing adequacy, treatment adherence, renin response, and individual aldosterone burden [91]. Moreover, real-world studies reveal that suboptimal MR antagonist dosing remains prevalent, leading to incomplete suppression of aldosterone’s pathological effects and potentially attenuated cardioprotective benefits [92].

Importantly, echocardiographic and cardiac magnetic resonance (CMR) findings synthesized in this review demonstrate that adrenalectomy more consistently reduces left ventricular mass index (LVMI), normalizes diastolic parameters (e.g., E/e′ ratio, left atrial volume index), and improves myocardial deformation indices such as global longitudinal strain (GLS) [26,29,66,85]. Beyond GLS, recent echocardiographic advances, particularly non-invasive pressure–strain loop analysis, have introduced myocardial work indices as load-adjusted parameters of myocardial efficiency. In PA, these indices reveal increased global wasted work and reduced myocardial work efficiency, which correlate with aldosterone-induced fibrosis and are partially reversible after adrenalectomy [29,51].

Advanced CMR techniques such as T1 mapping and late gadolinium enhancement (LGE) provide complementary tissue-level insight by detecting both focal and diffuse myocardial fibrosis, even in normotensive or mildly hypertensive PA patients, thereby underscoring the importance of early detection and intervention [85]. Structural remodeling captured on imaging aligns with clinical outcomes, including reduced incidence of heart failure and atrial fibrillation following successful adrenalectomy [29,93,94].

The pathogenesis of PA cardiomyopathy is now increasingly recognized as multifactorial, involving both traditional determinants, such as disease duration, degree of hypertension, sodium intake, and aldosterone exposure, and emerging modulators including oxidative stress, inflammatory biomarkers, endothelial dysfunction, genetic variants such as KCNJ5 mutations, and cortisol co-secretion [19,28,37,95]. Notably, in patients harboring KCNJ5 mutations, more pronounced and rapid regression of target organ damage has been observed following surgical intervention [37], underscoring the relevance of early genotypic identification in therapeutic decision-making and prognostication.

This evolving understanding paves the way for a more individualized therapeutic approach. Looking forward, the advent of nonsteroidal MR antagonists (e.g., finerenone) and aldosterone synthase inhibitors (e.g., Osilodrostat and Dexfadrostat) holds promise in enhancing the safety and specificity of medical therapy, potentially reducing off-target effects and improving adherence, particularly in patients with bilateral disease or those unsuitable for surgery [96,97,98,99]. However, their impact on cardiac remodeling in PA remains to be fully elucidated.

In conclusion, growing evidence supports adrenalectomy as the most effective intervention for reversing cardiac remodeling in patients with unilateral PA, while optimized medical therapy remains essential for those with bilateral disease. However, the evolving landscape of multimodality imaging, particularly the integration of speckle-tracking echocardiography and cardiac magnetic resonance (CMR) with T1 mapping and late gadolinium enhancement, offers unprecedented opportunities for early detection, precise phenotyping, and longitudinal monitoring of aldosterone-induced myocardial alterations. Future prospective, multicenter, and randomized studies are warranted to determine the prognostic relevance of residual LV hypertrophy and diffuse fibrosis as assessed by advanced imaging, validate myocardial work indices and deformation parameters in risk stratification, and identify imaging-guided biomarkers to tailor treatment strategies. Bridging these gaps will be pivotal to translating imaging-derived insights into precision medicine and improving cardiovascular outcomes in primary aldosteronism.

## 9. Summary of Findings, Clinical Implications, and Future Directions

This narrative review consolidates the current evidence indicating that adrenalectomy leads to superior and more consistent regression of left ventricular mass (LVM) compared to medical treatment with mineralocorticoid receptor antagonists (MRAs), particularly in patients with unilateral primary aldosteronism (PA) [21]. The surgical approach not only accelerates reverse cardiac remodeling but also offers significant and measurable improvements in diastolic and systolic function and cardiac volumes, reflecting a more complete restoration of cardiac structure and function [13,17,21]. These benefits appear to be magnified in subgroups with unilateral PA or those harboring aldosterone-producing adenoma with somatic KCNJ5 gene mutations, emphasizing the clinical value of early subtype diagnosis, multi-omics profiling, and immunohistochemical characterization, aiding in more accurate diagnosis and tailored treatment strategies [37]. Although MRAs remain indispensable for the management of bilateral adrenal hyperplasia or inoperable cases, their cardioprotective efficacy is highly dose-dependent and strongly contingent on achieving renin normalization, a goal often unmet in real-world practice due to subtherapeutic dosing or lack of standardized renin-based titration [91,92]. Therefore, the full potential of medical therapy is frequently underrealized. Moreover, cortisol co-secretion and genetic variability represent emerging determinants that modulate treatment response, and their under-recognition may compromise long-term cardiovascular protection [28,37].

In this complex pathophysiological context, echocardiography, particularly transthoracic echocardiography (TTE), represents a cornerstone for the serial assessment of PA-cardiomyopathy. Advanced TTE techniques, including tissue Doppler imaging (TDI) and speckle-tracking echocardiography (STE), enable non-invasive, real-time quantification of cardiac geometry, systolic and diastolic function, and strain parameters. These modalities are instrumental for detecting subclinical left ventricular dysfunction and early remodeling in PA, often preceding overt structural changes or symptomatic heart failure [100,101].

The complexity of PA-induced cardiomyopathy warrants a multimodal assessment that integrates advanced imaging, such as cardiac magnetic resonance (CMR), with T1 mapping and late gadolinium enhancement (LGE), to capture diffuse fibrosis and subclinical remodeling not visible on conventional echocardiography [8,85]. From a translational standpoint, this review underscores the urgent need to adopt a pathophysiologically tailored approach to therapy selection, reinforced by biomarker guidance and precise imaging phenotyping. Future research must address the heterogeneity in study protocols, therapy dosing, and follow-up durations by conducting multicenter prospective trials comparing surgical and optimized medical strategies with standardized cardiac endpoints. Additionally, the safety, efficacy, and long-term cardiac effects of novel nonsteroidal MR antagonists and aldosterone synthase inhibitors, currently under investigation in PA and resistant hypertension, must be rigorously evaluated. These agents hold promise for overcoming the limitations of classical MRAs and expanding therapeutic options, especially in bilateral disease. Importantly, the recent development of the Primary Aldosteronism Medical Treatment Outcome (PAMO) criteria offers a standardized framework to assess biochemical and clinical responses to medical therapy and will be instrumental in guiding future outcome-based research and clinical management [102]. Ultimately, integrating genetics, advanced imaging, including echocardiography, and molecular markers into clinical decision-making could mark a paradigm shift toward precision medicine in endocrine hypertension. This evolving evidence base calls for the refinement of existing clinical guidelines to prioritize early detection [103], subtype-specific management, and organ protection strategies in PA patients at heightened cardiovascular risk.

## Figures and Tables

**Table 1:** Imaging Characteristics of Primary Aldosteronism-Associated Cardiomyopathy.

Feature	Doppler and Speckle-Tracking Echocardiography	Cardiac Magnetic Resonance (CMR)
**Structural Remodeling**	Concentric remodeling and hypertrophy; increased LV mass index (LVMI), inappropriate excessive LVMI (ieLVMI), and predicted LVMI (pLVMI)	Increased LV mass; elevated extracellular matrix index (ECMi)
**Chamber Volumes**	Enlarged left atrial volume index (LAVi); elevated LV end-diastolic volume index (EDVi)	LV volume enlargement
**Diastolic Dysfunction**	Impaired filling: reduced E/A ratio (<1), increased E/e′ ratio, enlarged LA volume	Subclinical dysfunction: reduced peak diastolic strain rate, elevated filling volumes
**Systolic Function**	Preserved EF (>50%), but reduced global longitudinal strain (GLS) indicates subclinical dysfunction; myocardial work analysis shows increased global wasted work and reduced efficiency	EF typically >55%; subtle systolic dysfunction detectable by strain imaging
**Fibrosis Detection**	Indirect indicators only (e.g., wall thickening, chamber stiffness); no direct tissue characterization	LGE detects mid-wall patchy, non-infarct fibrosis; T1 mapping and ECV quantify diffuse fibrosis
**Tissue Characterization**	Limited	High-resolution myocardial assessment (LGE, native T1 mapping, ECV quantification)
**Myocardial Work** **Efficiency**	Increased wasted work and reduced efficiency	Under investigation

Comparative Echocardiographic and Cardiac MRI Features in Primary Aldosteronism versus Essential Hypertension and Data from PA-Only Studies. PA, Primary Aldosteronism; EH, Essential Hypertension; LVMI, Left Ventricular Mass Index; ieLVMI, Inappropriate Excessive LV Mass Index; pLVMI, Predicted LV Mass Index; ECMi, Extracellular Matrix Index; LAVi, Left Atrial Volume Index; EDVi, End-Diastolic Volume Index; EF, Ejection Fraction; GLS, Global Longitudinal Strain; LGE, Late Gadolinium Enhancement; T1, Native T1 Relaxation Time; ECV, Extracellular Volume Fraction.
